# Severe affective anesthesia presenting to the psychiatrist after surgical treatment for temporal lobe epilepsy

**DOI:** 10.1192/j.eurpsy.2026.11619

**Published:** 2026-07-31

**Authors:** R. L. D. Dios, J. de Sousa, M. R. Pinto, C. Campos, J. Mesquita

**Affiliations:** Hospital de Braga, Braga, Portugal

## Abstract

**Introduction:**

The role of amygdala in processing emotional expressions and emotional memory is well-established. Several neuroimaging studies have demonstrated that the amygdala is activated when individuals view emotional stimuli, such as movies. Furthermore, these studies have found a correlation between amygdala activity and the improvement in memory for emotional stimuli days later.

**Objectives:**

A case of affective anesthesia is presented, as a post-surgical complication, to our knowledge not previously reported in the literature, due to amygdala resection.

**Methods:**

We will describe a clinical case of a female patient, with no history of previous psychiatric follow-up, who presented to the psychiatrist after developing a condition of severe and sustained affective anesthesia directed towards her husband and three children, after undergoing a left anterior temporal lobectomy and amygdalohippocampectomy for the treatment of temporal lobe epilepsy (TLE) secondary to mesial sclerosis.

**Results:**

TLE is the most common form of drug-resistant epilepsy. Its exact cause is not yet fully understood, but the hippocampus, especially concerning hippocampal sclerosis, is implicated in most proposed mechanisms. Surgical treatment typically involves the resection of mesial temporal structures, which may include the hippocampus, parahippocampal gyrus, and basolateral amygdala. Despite the potential benefits of this intervention, it is not without risks. Complications can occur in up to 17% of patients, with the most common being bacterial meningitis and some transient neurological deficits. Some described psychopathological effects include depressive episodes, psychosis and memory deficits. At no point is there a description of affective anesthesia as a post-surgical complication, despite the theoretical correlation of the intervened structures with emotional memory.

**Conclusions:**

Mesial temporal lobe epilepsy has limited articles in the literature that specifically address its surgical outcomes and complications. Clinical studies in patients with different types of brain lesions at the temporal lobe level have confirmed that damage to the amygdala underlies impairments in memory for emotional material compared to those with an intact amygdala. It is important to emphasize the difficulty of deciding to act surgically in the face of a progressive degenerative disease, such as TLE, knowing it’s not without risks and these ones are still not fully explored. Therefore, ongoing research is necessary to improve outcomes and minimize complications in this area. The case reported proved to be rare and shocking, considering a patient who previously had stable and satisfactory affective relationships in her life.

**Disclosure of Interest:**

None Declared

